# Cognitive Ability in Late Life and Onset of Physical Frailty: The Lothian Birth Cohort 1936

**DOI:** 10.1111/jgs.14787

**Published:** 2017-03-01

**Authors:** Catharine R. Gale, Stuart J. Ritchie, Cyrus Cooper, John M. Starr, Ian J. Deary

**Affiliations:** ^1^ Centre for Cognitive Ageing and Cognitive Epidemiology Department of Psychology University of Edinburgh Edinburgh UK; ^2^ MRC Lifecourse Epidemiology Unit University of Southampton Southampton UK; ^3^ Geriatric Medicine Unit University of Edinburgh Western General Hospital Edinburgh UK

**Keywords:** fried frailty phenotype, processing speed, memory, visuospatial ability, crystallized ability

## Abstract

**Objectives:**

To investigate whether poorer cognitive ability is a risk factor for development of physical frailty and whether this risk varies according to cognitive domain.

**Design:**

Prospective longitudinal study with 6‐year follow‐up.

**Setting:**

Edinburgh, Scotland.

**Participants:**

Members of the Lothian Birth Cohort 1936 (N = 594).

**Measurements:**

Frailty was assessed at ages 70 and 76 using the Fried criteria. Cognitive function was assessed at age 70, 73, and 76. Factor score estimates were derived for baseline level of and change in four cognitive domains: visuospatial ability, memory, processing speed, and crystallized cognitive ability.

**Results:**

Higher baseline levels of processing speed, memory, visuospatial ability and crystallized ability at age 70, and less decline in speed, memory, and crystallized ability were associated with less risk of becoming physically frail by age 76. When all cognitive domains were modelled together, processing speed was the only domain associated with frailty risk, for a standard deviation (SD) increment in initial level of processing speed, the risk of frailty was 47% less (0.53 95% confidence interval (CI) = 0.33–0.85) after adjustment for age, sex, baseline frailty status, social class, depressive symptoms, number of chronic physical diseases, levels of inflammatory biomarkers, and other cognitive factor score estimates; for a SD increment in processing speed change (less decline) risk of frailty was 74% less (RRR = 0.26, 95% CI = 0.16–0.42). When additional analyses were conducted using a single test of processing speed that did not require fast motor responses (inspection time), results were similar.

**Conclusions:**

The speed with which older adults process information and the rate at which this declines over time may be an important indicator of the risk of physical frailty.

Frailty is a clinical syndrome observed in older adults, the core feature of which is greater vulnerability to stressors due to impairments in multiple systems, lower physiological reserves, and a decline in the ability to maintain homeostasis.^1^ It increases the risk of adverse outcomes.^1^, ^2^, ^3^ The phenotype model—in which frailty is based on three or more of five components: poor grip strength, slow walking speed, low physical activity, exhaustion, and unintentional weight loss^2^—is one of the two principal models of frailty.^1^ The frailty index, or cumulative deficit model, defines frailty in terms of the accumulation of deficits (symptoms, signs, diseases, disabilities), whereby an individual's frailty index score reflects the proportion of potential deficits present.^4^ These models differ in the potential role that cognitive impairment plays in their definition of frailty. The Fried phenotype defines frailty in purely physical terms, whereas the cumulative deficit model permits cognitive impairment to be included as a deficit. A consensus conference agreed that this broader definition of frailty should be distinguished from the medical syndrome of physical frailty.^5^ Given the importance of cognitive function and physical robustness for quality of life and survival, it is crucial to understand the extent to which cognitive ability and physical frailty are associated and the reasons for this.

Physical frailty and poorer cognitive function often coexist.^6^, ^7^, ^8^ The direction of this relationship and the underlying mechanisms are uncertain. Some longitudinal studies suggest that physical frailty increases risk of cognitive decline^9^, ^10^ or dementia.^11^, ^12^, ^13^ Poor cognitive function might be a risk factor for becoming physically frail, but evidence is sparse. Two longitudinal studies have found that lower Mini‐Mental State Examination (MMSE) scores increase the risk of incident physical frailty,^14^, ^15^ but it is unclear whether differences over the range of cognitive ability can predict the onset of physical frailty or whether some domains of cognitive ability are more important as risk factors than others. Results from a longitudinal study found that poorer executive function and greater decline in executive function were more strongly linked to physical frailty than level of or decline in psychomotor speed or memory.^16^ Further longitudinal investigations are needed to understand the role of specific cognitive domains in the development of physical frailty.

The Lothian Birth Cohort 1936 (LBC1936) was established to study cognitive aging.^17^ Three waves of data on processing speed, memory, visuospatial ability, and crystallized cognitive ability were used to examine how initial level of and change in cognitive function in these domains were related to risk of developing physical frailty or prefrailty.

## Methods

### Participants

The LBC1936 was established to study cognitive aging in surviving members of the 1947 Scottish Mental Survey.^17^, ^18^ Community‐dwelling people approximately age 70 were recruited (N = 1,091). Wave 2 took place when participants were approximately age 73 (n = 866). Wave 3 took place when participants were approximately age 76 (n = 697). Ethical approval was obtained from the Multi‐Centre Ethics Committee for Scotland and Lothian Research Ethics Committee.

### Measures

#### Physical Frailty

Frailty status was assessed during Waves 1 and 3 using the Fried phenotype,^2^ which defines frailty as the presence of three or more of unintentional weight loss, weakness, self‐reported exhaustion, slow walking speed, and low physical activity. Prefrailty is defined as the presence of one or two of these criteria. These criteria were operationalized using definitions similar to Fried's^2^, ^19^ (Appendix S1).

#### Cognitive Ability

Participants took a variety of cognitive tests in an identical fashion at each wave that were used as indicators of four domains of cognitive ability. Visuospatial ability was assessed according to scores on tests of matrix reasoning and block design from the Wechsler Adult Intelligence Scale (WAIS‐III^UK^)^20^ and spatial span forward and backward from the Wechsler Memory Scale (WMS‐III^UK^).^21^ Verbal‐declarative memory (henceforth memory) was assessed according to scores on tests of logical memory and verbal paired associates from the WMS‐III^UK^, and digit span backward from the WAIS‐III^UK^. Processing speed (henceforth speed) was assessed according to scores on tests of digit‐symbol substitution and symbol search from the WAIS‐III^UK^ and measures of four‐choice reaction time^22^ and inspection time.^23^ Of these measures of speed, inspection time is the only test requiring no speeded responses., in other words, participants are not required to respond as fast as possible. Crystallized cognitive ability was measured using National Adult Reading Test^24^ and Wechsler Test of Adult Reading^25^ scores, The MMSE was used solely to identify participants with likely cognitive impairment or dementia. With the exception of three of the tests for processing speed that required fast motor responses, none of the tests relied on physical function.

#### Covariates

Age, socioeconomic status, smoking status, number of chronic physical diseases, depressive symptoms, and inflammatory biomarkers were chosen at Wave 1 as potential confounding variables. Assessment details are given in Appendix S2.

### Statistical Analysis

The cognitive tests were organized into four domains: visuospatial ability, memory, speed, and crystallized ability. An intercept factor (baseline level of the ability) and a slope factor (change in the ability across the three waves) were estimated within each grouping using latent growth curve modelling in a factors‐of‐curves format.^26^ Latent‐variable models reduce the influence of test‐specific measurement error by using the shared variance between the baseline levels and changes in observed scores on multiple cognitive tests to estimate latent (unobserved) variables of cognitive ability baseline and change. Factor models and score estimates, which used full‐information maximum likelihood estimation to use all the data in the full sample at each wave, were produced using Mplus v7.3 (Muthén & Muthén, Los Angeles, CA). Details of the factors‐of‐curves structural equation models and mean decline in the cognitive test scores over the three waves are given in Appendix S3 and Table S1.

Other analyses were performed in Stata version 13 (Stata Corp., College Station, TX). Multinomial logistic regression was used to calculate relative risk ratios (RRR) of prefrailty or frailty at age 76 according to a standard deviation (SD) increment in factor score estimates for baseline cognitive ability in each domain and change in cognitive ability in each domain from age 70 to 76, with adjustment for potential confounding factors. Relationships did not vary according to sex, so the data were pooled, and sex was adjusted for. To reduce potential bias due to attrition, all models included inverse probability weights that made the sample more representative of the cohort at baseline.^27^ Three of the speed factor tests required fast, accurate motor responses. The fourth, inspection time, required no speeded response. To test whether associations found with the speed factor were artefacts caused by overlap of components of the frailty phenotype measure—slow walking speed and exhaustion—with the motor aspects of three of these tests, models were estimated in which only inspection time baseline and slope were used as predictors. Finally, analyses were repeated excluding participants who scored less than 24 on the MMSE.^28^

## Results

Analyses were based on 594 participants with data on all variables of interest. People excluded because of attrition tended to be older; had poorer cognitive ability, more depressive symptoms, more chronic physical disease, and higher blood concentrations of c‐reactive protein (CRP) and fibrinogen; were more likely to smoke and less likely to have professional or managerial socioeconomic status; and met more criteria for frailty at age 70. There were no significant differences between those in the sample and those excluded because of missing baseline data, except in level of the cognitive factor “speed,” which was lower in the missing‐data group (Table S2).

By age 76, 47.0% of the participants were prefrail, and 14.3% were frail. (At age 70, equivalent figures were 45.5% and 4.9% respectively.) The increase in prevalence of frailty between these ages is similar to that found previously.^29^ Of those who were frail at age 76, the most common combination of frailty criteria was exhaustion with low activity or slow walking speed (both occurring in 76.1%). In the sample as a whole, the most common frailty indicator was low activity (30.3%).

Table 1 shows participant characteristics according to frailty status at age 76. Greater frailty at age 76 was associated with older age, more depressive symptoms, more chronic physical disease, being a current smoker, having higher blood concentrations of CRP, and meeting more criteria for frailty at age 70. Greater frailty at age 76 was also associated with lower baseline level of visuospatial ability, memory, speed, and crystallized ability and greater decline in memory and speed between ages 70 and 76.

**Table 1:** Characteristics of Study Sample at Age 70 According to Frailty at Age 76

Characteristics	Not Frail, n = 230	Prefrail, n = 279	Frail, n = 85	*P*‐Value for Difference^a^
Age, mean ± SD	69.4 ± 0.83	69.5 ± 0.81	69.7 ± 0.77	<.001
Depressive symptom score, median (IQR)	1 (0–2)	1 (0–2)	2 (1–3)	<.001
Number of frailty criteria, median (IQR)	0 (0–1)	0 (0–1)	2 (1–2)	<.001
Number of chronic diseases, median (IQR)	0 (0–1)	1 (0–1)	1 (1–2)	<.001
Fibrinogen, g/L, median (IQR)	3.1 (2.7–3.5)	3.2 (2.8–3.5)	3.2 (2.9–3.7)	.14
C‐reactive protein, mg/L, median (IQR)	1.5 (1.5–5)	3 (1.5–6)	4 (1.5–7)	.02
Female, n (%)	108 (47.0)	139 (49.8)	44 (51.7)	.70
Current smoker, n (%)	11 (4.78)	17 (6.09)	11 (12.9)	.03
Professional or managerial social class, n (%)	144 (62.6)	166 (59.5)	47 (55.3)	.48
Cognitive factor score estimates for baseline level, mean ± SD				
Visuospatial ability	0.32 ± 0.85	0.17 ± 0.84	−0.42 ± 0.92	<.001
Memory	0.24 ± 0.79	0.12 ± 0.80	−0.29 ± 0.82	<.001
Speed	0.46 ± 0.81	0.18 ± 0.76	−0.54 ± 1.01	<.001
Crystallized ability	0.23 ± 0.94	0.12 ± 0.93	−0.29 ± 1.07	<.001
Cognitive factor score estimates for slope, mean ± SD				
Visuospatial ability	−0.02 ± 0.50	−0.04 ± 0.51	−0.01 ± 0.56	.85
Memory	0.10 ± 0.68	−0.03 ± 0.75	−0.28 ± 0.82	<.001
Speed	0.23 ± 0.54	−0.01 ± 0.69	−0.42 ± 0.81	<.001
Crystallized ability	−0.02 ± 0.87	−0.03 ± 1.08	0.003 ± 0.66	.96

a From analysis of variance, Kruskal–Wallis, or chi‐square tests as appropriate.

SD = standard deviation; IQR = interquartile range.

Table 2 shows the relative risk ratios of incident prefrailty or frailty at age 76 according to a SD increment in factor score estimates for baseline level of cognitive ability in each domain. In models adjusted for age, sex, and number of frailty criteria at baseline, higher factor scores for speed were associated with lower risk of becoming prefrail. This association was attenuated and no longer significant after further adjustment for other covariates and for other cognitive factor score estimates. There were no significant associations between any of the other cognitive factor score estimate levels and risk of becoming prefrail. In initial models, having a higher level of speed or visuospatial ability (but not memory or crystallized ability) was associated with a significantly lower risk of becoming frail by age 76 (becoming frail per SD increment in cognitive factor score estimates: RRR = 0.24, 95% CI = 0.17–0.35 for speed; RRR = 0.63, 95% CI = 0.42–0.93 for visuospatial ability). Further adjustment in the models of frailty for the other potential confounding factors had only a small attenuating effect on these associations. In a final model with frailty as the outcome, all cognitive factor score estimates were examined simultaneously. In this model, processing speed was the only cognitive domain that was independently associated with risk of becoming frail (SD increment in speed: RRR = 0.53, 95% CI = 0.33–0.85). When changes in depressive symptoms, in chronic physical illnesses, and in inflammatory markers between Waves 1 and 3 were adjusted for in place of these measures at Wave 1, results were similar (SD increment in speed: RRR = 0.46, 95% CI = 0.28–0.77).

**Table 2:** Risk of Incident Physical Prefrailty and Frailty at Age 76 According to Baseline Level of Cognitive Function at Age 70

Cognitive Factor Score Estimate for Baseline Level, per Standard Deviation	Adjusted for Age, Sex, and Components of Frailty Present at Age 70		Further Adjusted for Depressive Symptoms, Chronic Physical Diseases, Social Class, Inflammatory Biomarkers, and Smoking Status at Age 70		Further Adjusted for Other Cognitive Factor Score Estimates	
	Prefrail	Frail	Prefrail	Frail	Prefrail	Frail
	Relative Risk Ratios (95% Confidence Interval)					
Visuospatial ability	1.01 (0.79–1.30)	0.63 (0.42–0.93)	0.99 (0.76–1.30)	0.64 (0.41–0.98)	1.05 (0.78–1.63)	0.81 (0.50–1.31)
Memory	1.03 (0.78–1.04)	0.81 (0.55–1.21)	1.04 (0.78–1.40)	0.81 (0.52–1.26)	1.01 (0.73–1.38)	0.86 (0.52–1.40)
Speed	0.66 (0.52–0.84)	0.24 (0.17–0.35)	0.84 (0.64–1.10)	0.49 (0.32–0.76)	0.82 (0.61–1.09)	0.53 (0.33–0.85)
Crystallized ability	1.10 (0.87–1.40)	1.06 (0.77–1.46)	1.14 (0.87–1.51)	1.21 (0.81–1.81)	1.15 (0.86–1.54)	1.40 (0.90–2.18)

All estimates are weighted to adjust for attrition since baseline.

Table 3 shows RRRs for incident prefrailty or frailty according to a SD increment in factor score estimates for the slope of the trajectory of cognitive ability in each domain between ages 70 and 76. Higher factor score estimates for change in speed and in visuospatial ability—indicating less decline—were associated with lower risk of becoming prefrail. No other cognitive domain was independently associated with prefrailty. In initial models, for a SD increment in cognitive factor change—indicating less decline—the risk of pre‐frailty was less by 56% in the case of speed (RRR= 0.44, 95% CI 0.32 ‐ 0.62), and by 24% in the case of visuospatial ability (RRR=0.76, 95% CI = 0.53 ‐ 0.98) were (RRR = 0.44, 95% CI = 0.32, 0.62 for speed; RRR = 0.76, 95% CI = 0.53–0.98 for visuospatial ability). The association between change in speed and risk of prefrailty changed little in subsequent models, but the association between change in visuospatial ability and risk of prefrailty ceased to be significant when adjusted for other cognitive factor score estimates. In initial models of frailty, higher factor score estimates for change in speed and memory—indicating less decline—were associated with lower risk (becoming frail per SD increment in cognitive factor change: RRR = 0.20, 95% CI = 0.13–0.32 for speed; RRR = 0.48, 95% CI = 0.33–0.70 for memory). Further adjustment for the other covariates had only a small attenuating effect. In the final model, higher estimate for change in speed was the only cognitive factor score estimate that remained significantly associated with lower risk of frailty (SD increment: RRR = 0.26, 95% CI = 0.16–0.42). When changes in depressive symptoms, chronic physical illnesses, and inflammatory markers between Waves 1 and 3 were adjusted for in place of these measures at Wave 1, the association between change in speed and risk of frailty was very similar (SD increment in speed: RRR = 0.28, 95% CI = 0.17–0.46).

**Table 3:** Risk of Incident Physical Prefrailty and Frailty at Age 76 According to Change in Cognitive Function (Slope) Between Age 70 and 76

Cognitive Factor Score Estimates for Slope, per Standard Deviation	Adjusted for Age, Sex, and Components of Frailty Present at Age 70		Further Adjusted for Depressive Symptoms, Chronic Physical Diseases, Social Class, Inflammatory Biomarkers, and Smoking Status at Age 70		Further Adjusted for Other Cognitive Factor Score Estimates	
	Prefrail	Frail	Prefrail	Frail	Prefrail	Frail
	Relative Risk Ratios (95% Confidence Interval)					
Visuospatial ability	0.72 (0.53–0.98)	0.65 (0.40–1.06)	0.71 (0.52–0.98)	0.65 (0.39–1.06)	0.82 (0.59–1.15)	0.95 (0.56–1.63)
Memory	0.80 (0.62–1.03)	0.48 (0.33–0.70)	0.79 (0.61–1.02)	0.49 (0.33–0.72)	0.93 (0.70–1.24)	0.75 (0.48–1.15)
Speed	0.44 (0.32–0.62)	0.20 (0.13–0.32)	0.47 (0.34–0.65)	0.22 (0.15–0.36)	0.50 (0.35–0.70)	0.26 (0.16–0.42)
Crystallized ability	0.93 (0.77–1.12)	0.91 (0.69–1.19)	0.92 (0.776 1.11)	0.90 (0.68–1.18)	0.93 (0.77–1.13)	0.92 (0.69–1.24)

All estimates are weighted to adjust for attrition since baseline.

Table 4 shows RRRs for incident prefrailty or frailty according to SD increments in baseline level and change in inspection time. Results were similar to those obtained using the speed factor estimates.

**Table 4:** Risk of Incident Physical Prefrailty and Frailty at Age 76 According to Baseline Level of and Change in Inspection Time Between Age 70 and 76

Inspection Time Baseline Level or Slope, per Standard Deviation	Adjusted for Age, Sex, and Components of Frailty Present at Age 70		Further Adjusted for Depressive Symptoms, Chronic Physical Diseases, Social Class, Inflammatory Biomarkers, and Smoking Status at Age 70		Further Adjusted for Other Cognitive Factor Score Estimates	
	Prefrail	Frail	Prefrail	Frail	Prefrail	Frail
	Relative Risk Ratios (95% Confidence Interval)					
Level	0.76 (0.62–0.93)	0.56 (0.42–0.76)	0.77 (0.63–0.934)	0.58 (0.43–0.79)	0.76 (0.62–0.93)	0.60 (0.44–0.83)
Slope	0.87 (0.63–1.21)	0.60 (0.38–0.97)	0.86 (0.62–1.20)	0.61 (0.38–0.79)	0.86 (0.61–1.20)	0.65 (0.40–1.01)

All estimates are weighted to adjust for attrition since baseline.

The analyses were repeated excluding those who scored less than 24 on the MMSE at all three waves (n = 27). Results were almost unchanged (data not shown).

A sensitivity analysis was performed with those who were physically robust at age 70 (n = 295). Effect sizes were very similar to those presented in Tables 2 and 3; speed was the only cognitive domain associated with frailty risk in the fully adjusted models (SD increment in baseline level of speed: fully adjusted RRR = 0.78, 95% CI = 0.53–1.14 for prefrailty; RRR = 0.24, 95% CI = 0.09–0.61 for frailty; SD increment in change in speed: RRR = 0.49, 95% CI = 0.31–0.79 for prefrailty; RRR = 0.23, 95% CI = 0.10–0.57 for frailty).

## Discussion

To the knowledge of the authors, only one study has examined the relationship between different cognitive abilities and onset of physical frailty. In 331 women from the Women's Health and Aging Study, higher initial level of and slower decline in executive function—assessed using a single test—were associated with lower risk of physical frailty.^16^ Participants were also assessed for psychomotor speed and immediate and delayed verbal memory—again using single tests. Higher scores for speed, delayed verbal memory only, and general cognitive performance were associated with lower risk, but there were no significant associations between rate of decline on any cognitive test other than the test of executive function and physical frailty risk. The measure used to assess executive function in that study (the Trail‐Making Test) may also reflect processing speed,^30^ conforming to the findings in the current analysis.

In the present study, initial level of and decline in memory and speed were associated with frailty risk. Speed seemed to be the more‐powerful predictor of physical frailty because it was associated with risk independent of covariates and other cognitive domains; for a SD increment in initial level of speed or change in speed (less decline), risk of frailty was 47% or 74% less, respectively. To check whether overlap between the speed of motor response required by some tests of processing speed and the slow walking speed or exhaustion components of the frailty phenotype might produce these associations, the analyses were repeated using the psychophysical inspection time test as the sole measure of processing speed; this test of speed of visual discrimination does not rely on physical reactions. Effect sizes using this single test were smaller than those obtained using the speed factor—for a SD increment in baseline level of or change in inspection time, risk of frailty was 40% or 35% lower, respectively, after full adjustment—but these results demonstrate that the link between processing speed and risk of frailty is not artifactual. Processing speed may be an early signal of impending limitations in a number of physical–mental domains, with some underlying shared causes. There is evidence that greater decline in processing speed is associated with greater decline in walking speed,^31^ and in the current cohort, decline in processing speed, as measured according to inspection time, was strongly correlated with decline in general cognitive ability.^32^

The mechanisms underlying associations between domains of cognitive ability, in particular speed, and risk of physical frailty remain unclear. Adjustment for covariates had modest attenuating effects. Neuropathology that has an adverse effect on cognitive function may also influence risk of physical frailty. Support for this comes from findings that rates of change in physical frailty and cognitive function were strongly correlated and that Alzheimer's disease pathology, macroinfarcts, and nigral neuronal loss were associated with prior rates of change in physical frailty and cognitive ability.^33^ Disruption of connectivity in white matter affects processing speed^34^, ^35^ and walking speed.^36^ Further investigation in this cohort could test whether this is the mechanism underlying these findings. Another explanation might be that some common biological process of cellular senescence underlies the associations.^37^ Cellular senescence is a stress response that occurs when cells are exposed to potentially oncogenic stimuli. Senescent cells appear with increasing frequency in older tissues. The secretion of proinflammatory cytokines, growth factors, and proteases that accompanies cellular senescence may be implicated in cognitive decline and physical frailty.^38^

Strengths of this study include the characterization of each domain of cognitive function over three waves, enabling how initial level and change were related to onset of physical frailty or prefrailty to be examined. Other strengths are the narrow age range, data on a range of potential confounding factors, and the fact that the sample was of both sexes. One limitation is that, for some individuals, decline in cognitive ability and onset of physical frailty will have begun before age 70, making it uncertain whether poorer cognitive ability predates later frailty or whether cognitive and physical health are declining together. The finding that slower processing speed was as predictive of frailty in the subset of participants who were physically robust as in the whole sample suggests that poorer cognitive ability may increase the risk of frailty. A second limitation is that, largely because of attrition, the analyses were based on 54% of participants in the baseline survey. Attrition can result in biased estimates if there are differences in likelihood of follow‐up related to exposure and outcome. In the analytical sample, higher baseline levels of processing speed were associated with lower risk of becoming physically frail. The risk of becoming physically frail was likely to have been higher in those lost to follow‐up because they tended to be in poorer health and were frailer at baseline (Table S2). Those lost to follow‐up (and those excluded because of missing data) also differ from the analytical sample in having lower levels of processing speed. The models were weighted to reduce potential bias due to attrition, but the results may underestimate the predictive power of processing speed regarding risk of physical frailty.

The speed with which older people process information and the rate at which this declines may be important indicators of the risk of becoming physically frail. More research into cognitive domain–specific associations and risk of physical frailty is needed to confirm the importance of different domains for predicting onset of frailty and elucidate the underlying mechanisms.

## Supporting information


**Table S1.** Slope means for each cognitive test across the three waves (age 70 to age 76).
**Table S2.** Characteristics at wave 1 of participants included and excluded from the analytical sample.

**Appendix S1.** Operationalising the Fried phenotype of frailty criteria.
**Appendix S2.** Assessment of covariates.
**Appendix S3.** ‘Factors of curves’ structural equation models of the cognitive ability test scores.

## References

[1] Clegg A , Young J , Iliffe S et al. Frailty in elderly people. Lancet 2013;381:752–762. 23395245 10.1016/S0140-6736(12)62167-9 PMC4098658

[2] Fried LP , Tangen CM , Walston J et al. Frailty in older adults: Evidence for a phenotype. J Gerontol A Biol Sci Med Sci 2001;56A:M146–M156. 10.1093/gerona/56.3.m146 11253156

[3] Boyd CM , Xue QL , Simpson CF et al. Frailty, hospitalization, and progression of disability in a cohort of disabled older women. Am J Med 2005;118:1225–1231. 16271906 10.1016/j.amjmed.2005.01.062

[4] Rockwood K , Mitnitski A . Frailty in relation to the accumulation of deficits. J Gerontol A Biol Sci Med Sci 2007;62A:722–727. 10.1093/gerona/62.7.722 17634318

[5] Morley JE , Vellas B , van Kan GA et al. Frailty consensus: A call to action. J Am Med Dir Assoc 2013;14:392–397. 23764209 10.1016/j.jamda.2013.03.022 PMC4084863

[6] Robertson DA , Savva GM , Coen RF et al. Cognitive function in the prefrailty and frailty syndrome. J Am Geriatr Soc 2014;62:2118–2124. 25370593 10.1111/jgs.13111

[7] Wu YH , Liu LK , Chen WT et al. Cognitive function in individuals with physical frailty but without dementia or cognitive complaints: Results from the I‐Lan Longitudinal Aging Study. J Am Med Dir Assoc 2015;16:899e9–e16. 10.1016/j.jamda.2015.07.013 26321467

[8] Arts MH , Collard RM , Comijs HC et al. Physical frailty and cognitive functioning in depressed older adults: Findings from the NESDO Study. J Am Med Dir Assoc 2016;17:36–43. 26341037 10.1016/j.jamda.2015.07.016

[9] Auyeung TW , Lee JSW , Kwok T et al. Physical frailty predicts future cognitive decline—a four‐year prospective study in 2737 cognitively normal older adults. J Nutr Health Aging 2011;15:690–694. 21968866 10.1007/s12603-011-0110-9 PMC12878988

[10] Boyle PA , Buchman AS , Wilson RS et al. Physical frailty is associated with incident mild cognitive impairment in community‐based older persons. J Am Geriatr Soc 2010;58:248–255. 20070417 10.1111/j.1532-5415.2009.02671.x PMC3150526

[11] Buchman AS , Boyle PA , Wilson RS et al. Frailty is associated with incident Alzheimer's disease and cognitive decline in the elderly. Psychosom Med 2007;69:483–489. 17556640 10.1097/psy.0b013e318068de1d

[12] Avila‐Funes JA , Carcaillon L , Helmer C et al. Is frailty a prodromal stage of vascular dementia? Results from the Three‐City Study. J Am Geriatr Soc 2012;60:1708–1712. 22985143 10.1111/j.1532-5415.2012.04142.x

[13] Gray SL , Anderson ML , Hubbard RA et al. Frailty and incident dementia. J Gerontol A Biol Sci Med Sci 2013;68A:1083–1090. 10.1093/gerona/glt013 PMC3738027 23419778

[14] Raji MA , Al Snih S , Ostir GV et al. Cognitive status and future risk of frailty in older Mexican Americans. J Gerontol A Biol Sci Med Sci 2010;65:1228–1234. 20622137 10.1093/gerona/glq121 PMC2954238

[15] Aranda MP , Ray LA , Snih SA et al. The protective effect of neighborhood composition on increasing frailty among older Mexican Americans: A barrio advantage? J Aging Health 2011;23:1189–1217. 21948774 10.1177/0898264311421961 PMC3506387

[16] Gross AL , Xue QL , Bandeen‐Roche K et al. Declines and impairment in executive function predict onset of physical frailty. J Gerontol A Biol Sci Med Sci 2016;71:1624–1630. 27084314 10.1093/gerona/glw067 PMC5106857

[17] Deary IJ , Gow AJ , Pattie A et al. Cohort profile: The Lothian Birth Cohorts of 1921 and 1936. Int J Epidemiol 2012;41:1576–1584. 22253310 10.1093/ije/dyr197

[18] Deary IJ , Gow AJ , Taylor MD et al. The Lothian Birth Cohort 1936: A study to examine influences on cognitive ageing from age 11 to age 70 and beyond. BMC Geriatr 2007;7:28. 18053258 10.1186/1471-2318-7-28 PMC2222601

[19] Bandeen‐Roche K , Xue QL , Ferrucci L et al. Phenotype of frailty: Characterization in the women's health and aging studies. J Gerontol A Biol Sci Med Sci 2006;61A:262–266. 10.1093/gerona/61.3.262 16567375

[20] Wechsler D . Wechsler Adult Intelligence Scale III‐UK Administration and Scoring Manual. London: Psychological Corporation, 1998.

[21] Wechsler D . Wechsler Memory Scale III‐UK Administration and Scoring Manual. London: Psychological Corporation, 1998.

[22] Deary IJ , Der G , Ford G . Reaction times and intelligence differences—a population‐based cohort study. Intelligence 2001;29:389–399.

[23] Deary IJ , Simonotto E , Meyer M et al. The functional anatomy of inspection time: An event‐related fMRI study. NeuroImage 2004;22:1466–1479. 15275904 10.1016/j.neuroimage.2004.03.047

[24] Nelson HE , Willison JR . National Adult Reading Test, 2nd Ed Windsor: NFER‐Nelson, 1991.

[25] Wechsler D . Wechsler Test of Adult Reading: WTAR. San Antonio: Psychological Corporation, 2001.

[26] McArdle JJ . Dynamic but structural equation modeling of repeated measures data In: Nesselroade JR, Cattell RB, eds. Handbook of Multivariate Experimental Psychology. New York: Springer, 1988, pp 561–614.

[27] Seaman SR , White IR . Review of inverse probability weighting for dealing with missing data. Stat Methods Med Res 2013;22:278–295. 21220355 10.1177/0962280210395740

[28] Folstein MF , Folstein SE , McHugh PR . ‘Mini‐mental state’. A practical method for grading the cognitive state of patients for the clinician. J Psychiatr Res 1975;12:189–198. 1202204 10.1016/0022-3956(75)90026-6

[29] Gale CR , Cooper C , Aihie Sayer A . Prevalence of frailty and disability: Findings from the English Longitudinal Study of Ageing. Age Ageing 2015;44:162–165. 25313241 10.1093/ageing/afu148 PMC4311180

[30] Salthouse TA . Relations between cognitive abilities and measures of executive functioning. Neuropsychology 2005;19:532–545. 16060828 10.1037/0894-4105.19.4.532

[31] Gale CR , Allerhand M , Sayer AA et al. The dynamic relationship between cognitive function and walking speed: The English Longitudinal Study of Ageing. Age 2014;36:9682. 24997019 10.1007/s11357-014-9682-8 PMC4119879

[32] Ritchie SJ , Tucker‐Drob EM , Deary IJ . A strong link between speed of visual discrimination and cognitive ageing. Curr Biol 2014;24:R681–R683. 25093556 10.1016/j.cub.2014.06.012 PMC4123160

[33] Buchman AS , Yu L , Wilson RS et al. Brain pathology contributes to simultaneous change in physical frailty and cognition in old age. J Gerontol A Biol Sci Med Sci 2014;69A:1536–1544. 10.1093/gerona/glu117 PMC4296120 25136002

[34] Penke L , Munoz Maniega S , Murray C et al. A general factor of brain white matter integrity predicts information processing speed in healthy older people. J Neurosci 2010;30:7569–7574. 20519531 10.1523/JNEUROSCI.1553-10.2010 PMC6632368

[35] Kuznetsova KA , Maniega SM , Ritchie SJ et al. Brain white matter structure and information processing speed in healthy older age. Brain Struct Funct 2016;221:3223–3235. 26254904 10.1007/s00429-015-1097-5 PMC4920858

[36] Rosario BL , Rosso AL , Aizenstein HJ et al. Cerebral white matter and slow gait: Contribution of hyperintensities and normal‐appearing parenchyma. J Gerontol A Biol Sci Med Sci 2016;71:968–973. 26755683 10.1093/gerona/glv224 PMC4906323

[37] Campisi J , d'Adda di Fagagna F . Cellular senescence: When bad things happen to good cells. Nat Rev Mol Cell Biol 2007;8:729–740. 17667954 10.1038/nrm2233

[38] LeBrasseur NK , Tchkonia T , Kirkland JL . Cellular senescence and the biology of aging, disease, and frailty. Nestle Nutr Inst Workshop Ser 2015;83:11–18. 26485647 10.1159/000382054 PMC4780350

