# Supplementary material for: Cognitive Ability in Late Life and Onset of Physical Frailty: The Lothian Birth Cohort 1936
Source: J Am Geriatr Soc. 2017 Mar 1;65(6):1289–95. doi: 10.1111/jgs.14787 (PMC5482391; doi:10.1111/jgs.14787)
Supplement: Supplementary file 1 — Table S1. Slope means for each cognitive test across the three waves (age 70 to age 76). Table S2. Characteristics at wave 1 of participants included and excluded from the analytical sample. [file JGS-65-1289-s001.docx]

Table S1: Slope Means for Each Cognitive Test Across the Three Waves (Age 70 to 76)

| Cognitive Domain and Test | b | Standard Error | P-Value |
| --- | --- | --- | --- |
| Visuospatial ability |  |  |  |
| Matrix reasoning | −0.156 | 0.023 | < .001 |
| Block design | −0.415 | 0.038 | < .001 |
| Spatial span | −0.027 | 0.007 | < .001 |
| Memory |  |  |  |
| Verbal paired associates | −0.197 | 0.043 | < .001 |
| Logical memory | 0.105 | 0.089 | .24 |
| Digit span backward | −0.023 | 0.011 | .03 |
| Speed |  |  |  |
| Symbol search | −0.149 | 0.029 | < .001 |
| Digit-symbol substitution | −0.703 | 0.047 | < .001 |
| Inspection time | −0.493 | 0.065 | < .001 |
| Choice reaction time | −0.071 | 0.004 | < .001 |
| Crystallized ability |  |  |  |
| National Adult Reading Test | −0.026 | 0.017 | .13 |
| Wechsler Test of Adult Reading | −0.079 | 0.016 | < .001 |

There was significant mean decline in all but two of the cognitive tests between the ages of 70 and 76; the tests of memory showed less decline, possibly because some of the participants recalled some of their content (which was identical wave-to-wave) from the previous wave. The tests of speed showed, on average, the largest declines across the waves.

Table S2: Characteristics at Wave 1 of Participants Included in and Excluded from the Analytical Sample

| Characteristic | Included in Analytical Sample | | | P for Difference Between Those in the Sample and Those Excluded^a^ | |
| --- | --- | --- | --- | --- | --- |
|  | Yes, n=594 | No: Attrition, n=394 | No: Missing Data, n=103^b^ | Because of Attrition | Because of Missing Data |
| Age, mean±SD | 69.5±0.82 | 69.6±0.84 | 69.6±0.88 | .047 | .17 |
| Depressive symptom score, median (IQR) | 1 (0–2) | 1 (0–3) | 1 (0–3) | .007 | .09 |
| Number of frailty criteria, median (IQR) | 1 (0–1) | 1 (0–2) | 0 (0–1) | <.001 | .85 |
| No of chronic diseases, median (IQR) | 1 (0–1) | 1 (0–2) | 1 (0–2) | .003 | .32 |
| Fibrinogen, g/L, median (IQR) | 3.2 (2.8–3.6) | 3.3 (2.9–3.8) | 3.3 (2.9–3.6) | .002 | .25 |
| C-reactive protein, mg/L, median (IQR) | 3 (1.5–5) | 4 (1.5–8) | 4 (1.5–7) | .001 | .05 |
| Female, % | 49.0 | 47.9 | 44.7 | .31 | .42 |
| Current smoker, % | 6.57 | 19.0 | 10.7 | <.001 | .13 |
| Professional or managerial socioeconomic class, % | 60.1 | 47.9 | 55.3 | <.001 | .38 |
| Cognitive factor score estimates for level, mean±SD |  |  |  |  |  |
| Visuospatial ability | 0.14±0.89 | –0.23±0.87 | 0.07±0.91 | <.001 | .47 |
| Memory | 0.11±0.82 | –0.19±0.78 | 0.10±0.93 | <.001 | .99 |
| Speed | 0.18±0.88 | –0.24±0.87 | –0.17±0.89 | <.001 | <.001 |
| Crystallized ability | 0.10±0.94 | –0.14±1.00 | –0.07±1.11 | <.001 | .11 |

^a^rom t-tests, Kruskal-Wallis tests, or chi-square tests as appropriate.

^2^Maximum number on which these analyses were based.

SD=standard deviation; IQR=interquartile range.
