# Supplementary material for: Cognitive Ability in Late Life and Onset of Physical Frailty: The Lothian Birth Cohort 1936
Source: J Am Geriatr Soc. 2017 Mar 1;65(6):1289–95. doi: 10.1111/jgs.14787 (PMC5482391; doi:10.1111/jgs.14787)
Supplement: Supplementary file 2 — Appendix S1. Operationalising the Fried phenotype of frailty criteria. Appendix S2. Assessment of covariates. Appendix S3. ‘Factors of curves’ structural equation models of the cognitive ability test scores. [file JGS-65-1289-s002.docx]

Appendix 1: Operationalizing the Fried Phenotype of Frailty Criteria

Height was measured using a portable stadiometer and weight using an electronic scale. Body mass index (BMI) was calculated as weight (in kilograms)/height (in meters)^2^. Weight loss was defined as current BMI less than 18.5 kg/m^2^ or, at Wave 3 only, loss of weight of 10% or more since the Wave 1 examination. Maximum handgrip strength was measured three times on each side using a dynamometer; the best of these measurements was used for analysis. Weakness was defined as maximum grip strength in the lowest 20% of the distribution, adjusted for sex and BMI. Exhaustion was considered present if the participant responded “very often” or “nearly all the time” to the statement “I feel as if I’m slowed down,” taken from the Hospital Anxiety and Depression Scale.^1^ Walking speed was assessed by measuring the time taken to walk a distance of 6 m at maximum speed. Slow walking speed was defined as a walking speed in the lowest 20% of the distribution, adjusted for sex and height. Participants were asked to indicate their usual level of physical activity on a 6-point scale, ranging from moving only in connection with necessary (household) chores to keeping fit, heavy exercise, or competitive sport several times a week. Low physical activity was defined as activity in the lowest sex-specific 20% of the distribution.

**References**

1. Zigmond AS, Snaith RP. The Hospital Anxiety and Depression Scale. Acta Psychiatr Scand 1983;67:361–370

**Appendix 2: Assessment of Covariates**

Socioeconomic position was derived from the participant’s highest reported occupation (or their spouse’s if it was higher) and classified into five social class categories: professional, managerial, skilled nonmanual, skilled manual, and semiskilled or unskilled. Participants provided information during an interview as to whether they had been diagnosed with diabetes mellitus, stroke, cardiovascular disease, high blood pressure, or cancer; a variable was derived for number of chronic physical diseases. Participants provided information in the interview as to whether they were current smokers, exsmokers, or had never smoked. Symptoms of depression were assessed using the depression subscale of the Hospital Anxiety and Scale^1^. Because one item from this subscale—I feel as if I’m slowed up—was used as an indicator of exhaustion when deriving the frailty phenotype, this item was excluded when total depression score was calculated. Blood samples were taken for the measurement of C-reactive protein and fibrinogen.

**References**

1. Zigmond AS, Snaith RP. The Hospital Anxiety and Depression Scale. Acta Psychiatr Scand 1983;67:361–370

**Appendix 3: Factors-of-Curves Structural Equation Models of the Cognitive Ability Test Scores**

To estimate the factors-of-curves model, individual latent growth curve models across the three testing waves (approximate mean age 70, 73, 76) were estimated for each of the cognitive tests, and the resulting latent intercept and slope factors for each test were used as indicators of higher-order domain intercepts and slopes. (Thus, the individual test growth curves were factor-analyzed.) For example, the latent intercepts and latent slopes for matrix reasoning, block design, and spatial span (forwards and backwards) were used as indicators of the higher-order level and slope of visuospatial ability (mutatis mutandis for the remaining three cognitive domains). The parameters of the slope factor for each test were set to the precise gap in years between each testing wave (2.98 years on average between Waves 1 and 2; 6.75 years on average between Waves 1 and 3).

The results reported in the main document come from growth curves with their intercept set at the final wave (such that the cognitive decline slope occurred before the intercept), although estimating the growth curves with their intercept at the first wave made little difference in the results. The domain factors extracted are reliable indices of each participant’s cognitive baseline for that domain and the extent of their cognitive change in it from age 70 to 76. The factor score estimates were extracted from the models and used in subsequent models predicting frailty status. Some slopes had residual variances estimated as negative because they were near zero; they were fixed to zero, and it was thus assumed that all of their variance was associated with the general factor of slope.

The final model that was used to estimate the factor scores had excellent fit to the data (visuospatial: χ^2^(54) = 168.24, p < .001, root mean square of approximation (RMSEA) = 0.044, comparative fit index (CFI) = 0.976, Tucker-Lewis Index (TLI) = 0.970; memory: χ^2^(27) = 128.16, p < .001, RMSEA = 0.059, CFI = 0.975, TLI = 0.967; speed: χ^2^(56) = 158.22, p < .001, RMSEA = 0.041, CFI = 0.985, TLI = 0.982; crystallized cognitive ability: χ^2^(28) = 68.29, p < .001, RMSEA = 0.036, CFI = 0.996, TLI = 0.995). That is, the cognitive predictor variables used were a good fit to the three waves of data in the study.
